# Metabotropic Glutamate Receptor 3 Expression During Liver Disease Progression: Association with Inflammation and Cell Viability in Hepatocellular Carcinoma

**DOI:** 10.3390/ijms27093878

**Published:** 2026-04-27

**Authors:** Ana Cristina García-Gaytán, Andy Hernández-Abrego, Dalia De Ita-Pérez, Ericka de los Ríos-Arellano, Emanuel Gámez, Mauricio Díaz-Muñoz, Isabel Méndez

**Affiliations:** 1 Departamento de Neurobiología Celular y Molecular, Instituto de Neurobiología, Universidad Nacional Autónoma de México (UNAM), Campus UNAM-Juriquilla, Juriquilla 76230, Querétaro, Mexicoandy.bha@comunidad.unam.mx (A.H.-A.); daludek@hotmail.com (D.D.I.-P.); erios.histologia.inb@gmail.com (E.d.l.R.-A.); mdiaz@comunidad.unam.mx (M.D.-M.); 2 Centro Médico Nacional de Occidente, Instituto Mexicano del Seguro Social, Guadalajara 44340, Jalisco, Mexico; dremanuelgamez@hotmail.com

**Keywords:** glutamate, metabotropic glutamate receptor type 3, inflammation, fibrosis, cirrhosis, hepatocellular carcinoma

## Abstract

Hepatocellular carcinoma (HCC) is the most common type of liver cancer that is mostly preceded by cirrhosis, with a high mortality rate. Therefore, diagnosis is critical in the early stages. In this study, we explored the liver expression of metabotropic glutamate receptor 3 (mGluR3), a group II mGluR, during the progression from fibrosis to cirrhosis and, ultimately, to HCC induced by diethylnitrosamine (DEN) in rats. We found that mRNA expression of mGluR3 (*Grm3*) was upregulated in HCC, while the protein level was significantly increased from the cirrhosis stage, and even more in HCC. *Grm3* correlated with interleukin-6 (*Il6*) and transforming growth factor-β (*Tgfb*) mRNA expression. Furthermore, serum and intrahepatic glutamate concentrations were augmented in HCC. Immunohistochemical analysis revealed that mGluR3 is expressed in hepatocytes and non-parenchymal cells (endothelial cells and macrophages), and we observed a positive signal in the cytoplasmic membrane, cytoplasm, and nuclei of tumor and non-tumor cells. We confirmed that normal hepatocytes (C9 cell line) express low levels of mGluR3 protein and HCC-derived cells (HepG2) express high levels of this receptor. Using HepG2 cells, we observed that mGluR3 activation by glutamate and the group II-selective agonist LY354740 treatments were functional, as both inhibited cAMP generation induced by forskolin and increased cellular viability with no effect on dead cells. These results showed that mGluR3 is differentially expressed throughout the progression of liver pathologies, is associated with the inflammatory environment, and plays a role in HCC cell survival, with potential utility as an early biomarker and therapeutic target.

## 1. Introduction

Glutamate is the major excitatory neurotransmitter in the brain and one of the most abundant amino acids in the blood. Its plasma concentrations are in the range of 50–100 mM [1]. This amino acid functions as both a biochemical intermediate and a messenger, since it mediates cellular responses through its binding to two classes of membrane receptors: ion channels, or ionotropic glutamate receptors (iGluRs), and G-protein-coupled receptors or metabotropic glutamate receptors (mGluRs) [2]. According to their pharmacology, amino acid sequence homology, and signal transduction pathways, mGluRs are classified into eight subtypes (mGluR1–8 and their genes GRM1–8) and divided into three groups: group I (mGluR1 and mGluR5), group II (mGluR2 and mGluR3), and group III (mGluR4, mGluR6–8) [3]. Aside from their neuromodulatory roles in the central nervous system, mGluRs have been implicated in the pathophysiology of cancer in neural and non-neural cells [4,5,6,7]. Particularly, mGluR3 is expressed or overexpressed in different cancer cell types, such as melanoma, breast, colon, prostate, and lymphoma [8], and/or has implications for tumor cell features. For example, aldosterone-producing adenomas have the highest mGluR3 expression compared to cortisol-producing adenomas and normal adrenal cortex tissues. This receptor participates in steroidogenesis in adrenocortical tissues by enhancing aldosterone/cortisol production [9]. In breast cancer, mGluR3 activation promotes basement membrane disruption and invasiveness through an autocrine action of glutamate [4]. mGluR3 is upregulated in various human colon cancer cell lines [8], and its knockdown expression diminishes cell survival and tumor growth [7], while mutations of this receptor found in melanoma contribute to cell growth and migration [10].

Hepatocellular carcinoma (HCC) is a primary tumor and constitutes the most common type of liver cancer, with a high prevalence in men [11]. According to Globocan (2022), HCC is a major cause of liver cancer deaths and the fourth most common cause of cancer worldwide, with a 1:1 mortality-to-incidence ratio (https://gco.iarc.who.int (accessed on 22 April 2026)). Research has predicted that the incidence of this cancer type will rise by >55% by 2040 [12]. It is currently accepted that HCC arises as a progression from chronic liver disease to cirrhosis [13]. HCC etiology includes chronic hepatitis B or C, alcohol abuse, and metabolic dysfunction–associated steatotic liver disease or MASLD [14]. These causal agents of hepatocyte injury, accompanied by an inflammatory response, favor the installation of hepatic fibrosis, cirrhosis, and HCC [15,16]. Conducting molecular analysis of tissue or liquid biopsies is relevant for better diagnosis and treatment, as there are still no reliable non-invasive methods available. Understanding the molecular alterations that precede the establishment of HCC could enhance early-stage detection tools and enable more effective treatment with fewer secondary effects.

Little is known about mGluR3 expression in the liver, both in normal cells and in HCC, and about its possible role in normal function or in the pathophysiology of liver diseases. A unique study reported the presence of mGluR3 protein in CCl_4_-induced cirrhotic liver in rats, particularly in macrophages located in the fibrous septa [17]. Additionally, higher glutamate concentrations have been found in patients with liver diseases, such as hepatitis and HCC, compared with healthy subjects [18]. The liver is a main source of blood glutamate [19]. It has been shown that patients with HCC exhibit high expression of antiporter xCT mRNA in the liver, which provides extracellular glutamate and correlates with poor overall and disease-free survival [20]. Therefore, we hypothesized that extracellular glutamate is exported from intrahepatic glutamate because of the overexpression of the transporter, thereby enabling mGluR3 activation. Thus, we analyzed the expression of the xCT antiporter and its counterpart, the glutamate uptake transporter, in the progression of liver pathologies.

Based on this knowledge, this study aimed to analyze the expression of mGluR3 in the liver using a well-established experimental model of the sequential pathologies of fibrosis, cirrhosis, and HCC in rats, and corroborate the findings in the cancerous HepG2 cell line and the normal hepatocyte C9 cell line. We also investigated the possible role of mGluR3 in cell viability in culture by treating cells with glutamate or a selective mGluR3 agonist in a glutamate-free culture medium.

## 2. Results

### 2.1. Progression of Hepatic Abnormalities upon DEN Treatment

**Figure 1:**
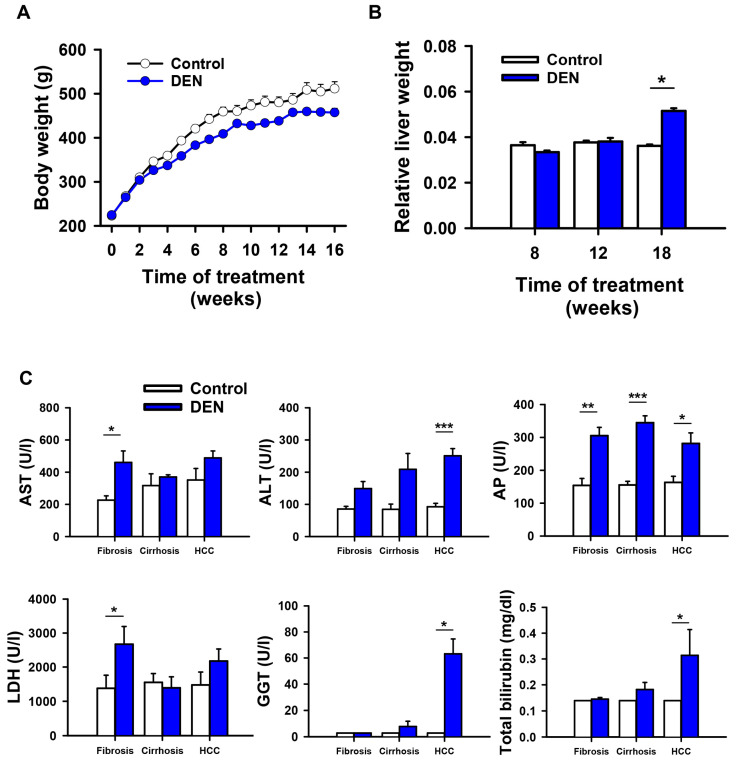
Physical and biochemical characteristics of liver pathological stages induced by DEN in rats. (**A**) Body weight and (**B**) liver weight normalized to body weight during 16 weeks of weekly DEN administration. (**C**) Hepatic enzymes in the serum of DEN-treated rats at weeks 8, 12, and 18. AST: aspartate transaminase, ALT: alanine transaminase, AP: alkaline phosphatase, LDH: lactate dehydrogenase, GGT: gamma-glutamyl transpeptidase. Graphs represent mean ± SEM, *n* = 4–5 rats. * *p* < 0.05, ** *p* < 0.01, *** *p* < 0.001 by *t*-test.

**Figure 2:**
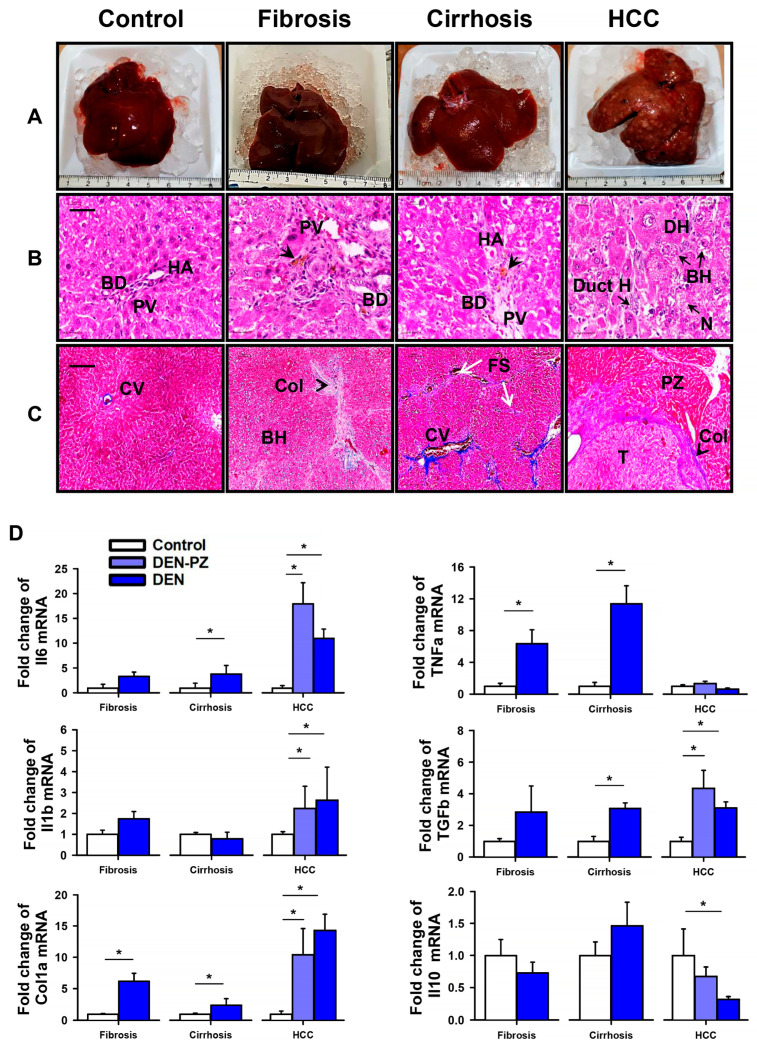
Histopathological changes and markers for inflammation in DEN-treated rats. Promotion of fibrosis and inflammation by DEN. (**A**) Representative macroscopic images of liver sections during the progression of liver pathologies. (**B**) Hematoxylin and eosin-stained livers and (**C**) Masson’s Trichrome-stained livers. Dysplastic lesions and neoplastic lesions in cirrhosis and HCC are observed. Scale bar 50 mm. Identification tags: BD: bile duct, BH: balloon hepatocyte, Col: collagen, Duct H: ductular hepatocyte, FS: fibrous septa, HA: hepatic artery, N: necrosis, PV: portal vein, PZ: peritumoral zone, T: tumor. Arrows indicate hemosiderin deposits. (**D**) Inflammation and fibrosis markers during the progression of the liver pathologies. Relative mRNA expression of Interleukin-6 (*Il6*), Tumor Necrosis Factor-α (*Tnfa*), Transformed Growth Factor-β (*Tgfb*), Interleukin 1-b (*Il1b*), collagen 1a and Interleukin-10 (*Il10*) in the liver of control and experimental rats determined by qRT-PCR. Data were normalized to the expression of Rps18 mRNA. In hepatocarcinoma (HCC), the light blue bar indicates the peritumoral zone. Graphs represent mean ± SEM, *n* = 3–13 samples from three different experiments, * *p* < 0.05, by *t*-test or one-way ANOVA for parametric data and Mann–Whitney U or Kruskal–Wallis for non-parametric data.

Inducing hepatic damage with DEN in rats is a well-established model that reproduces biochemical, histological, and genetic features similar to those observed in humans. This damage progresses over three stages: liver fibrosis by week 8, cirrhosis by week 12, and HCC by week 18 [21,22,23]. In this study, we observed the sequential characteristics of each pathological stage during the 18 weeks of DEN treatment. The body weight of experimental and control rats was registered weekly throughout the experiment. We found that weight gain decreased (~10%) in DEN-treated rats from week 6 when compared to control rats, which were injected weekly with saline solution (Figure 1A). During tissue dissection, we observed that DEN administration altered liver consistency. These alterations were associated with the tissue fibrosis that occurs during liver disease progression and hepatomegaly (Figure 1B). Additionally, we observed the growth of preneoplastic and neoplastic lesions in week 18, which is consistent with the HCC stage (Figure 2A). Liver dysfunction was confirmed by increased serum levels of biomarkers at the different stages of liver pathology. These biomarkers included AST, ALT, AP, LDH, GGT, and total bilirubin (Figure 1C). Analysis by MRI showed the development of cancerous nodules in the rat livers during the progression from normal tissue to tissue with cirrhosis and HCC (Supplementary Figure S1).

The histopathological analysis of liver tissues revealed normal architecture in the control group, with portal triads, well-structured hepatocyte cords, a conserved endothelium, and little presence of collagen around the veins (Figure 2B,C). We also observed collagen deposits and inflammatory cell infiltration from the fibrotic stage, along with an increase the fibrosis marker collagen type I, consistent with the presence of fibrosis across all stages of liver damage (Figure 2B–D). Using Masson’s trichrome staining, we also identified extensive fibrous septae corresponding to cirrhosis and hemosiderin deposits at week 12 of the experimental protocol (Figure 2C). Foci of cellular alteration (dysplastic nodules) and neoplastic lesions were observed starting from the cirrhosis stage and were also present in liver sections with HCC. Furthermore, the HCC sections exhibited a loss of architecture, basophilic foci, ballooned hepatocytes in the lobular zone, and ductular hepatocytes (Figure 2B,C). Liver mRNA expression of pro-inflammatory cytokines *Il1b*, *Il6*, and *Tnfa* and the anti-inflammatory and pro-fibrotic cytokine TGF-b (*TGFb*) increased at different stages of liver pathology, while anti-inflammatory cytokine *Il10* diminished in HCC, as previously reported [24]. 

### 2.2. Hepatic Glutamate and Its Transporters in the Liver Are Modified in HCC

**Figure 3:**
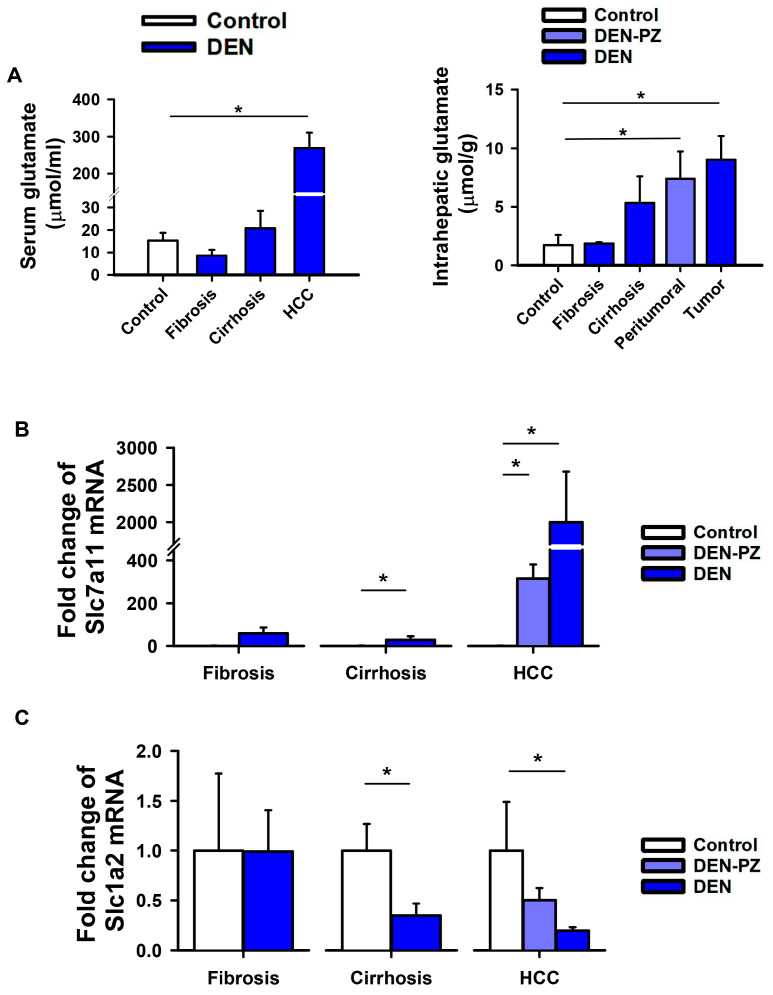
Circulating and hepatic glutamate levels, and gene expression of liver glutamate transporters in liver pathologies induced by DEN. (**A**) Increased serum and intrahepatic glutamate levels were observed during the progression of the liver pathologies analyzed by enzymatic assays. Graphs represent mean ± SEM. (*n* = 3–4). * *p* < 0.05 by one-way ANOVA, Dunn and Tukey post hoc tests, respectively. Changes in gene expression of glutamate transporters (**B**) xCT (*Slc7a11*) and (**C**) EAAT2 (*Slc1a2*) during the progression of the liver pathologies, as analyzed by RT-qPCR in livers from control and experimental rats. In HCC, DEN-PZ indicates the peritumoral zone. Graphs represent mean ± SEM (*n* = 3–12 samples from three different experiments). * *p* < 0.05, by *t*-test or ANOVA and Dunn’s post hoc test.

Increased plasma glutamate concentrations have been reported in patients with liver diseases [18]. We investigated serum and intrahepatic glutamate concentrations in the rat liver across the pathological stages induced by DEN. Serum glutamate concentrations were significantly higher in HCC, and the intrahepatic content of glutamate increased significantly in neoplastic tissue, in both the tumor and peritumoral zones (Figure 3A). Research has shown that glutamate can be transported through the cytoplasmic membrane via transporters. System xCT (*Slc7a11* gene) mediates the exchange of intracellular glutamate for extracellular cystine and is overexpressed in several cancer types, such as HCC, thereby contributing to cell growth [25]. Excitatory amino acid transporter 2 (EAAT2, *Slc1a2* gene) is expressed in perivenous mouse hepatocytes [26]. In this study, we measured the mRNA expression of both transporters during the progression of the DEN-induced liver pathologies. Our findings indicate that *Slc7a11* increased significantly during the cirrhosis stage and even more in HCC in the tumor and peritumoral zones (Figure 3B). In contrast, *Slc1a2* decreased during cirrhosis and continued to decrease during HCC in the tumor and peritumoral zones (Figure 3C).

These observations suggest that liver glutamate is preferentially exported and could be available to bind and activate glutamate receptors, such as mGluR3.

### 2.3. Grm3 mRNA Is Upregulated in HCC and Correlates with Il6 and Tgfb mRNA Expression

**Figure 4:**
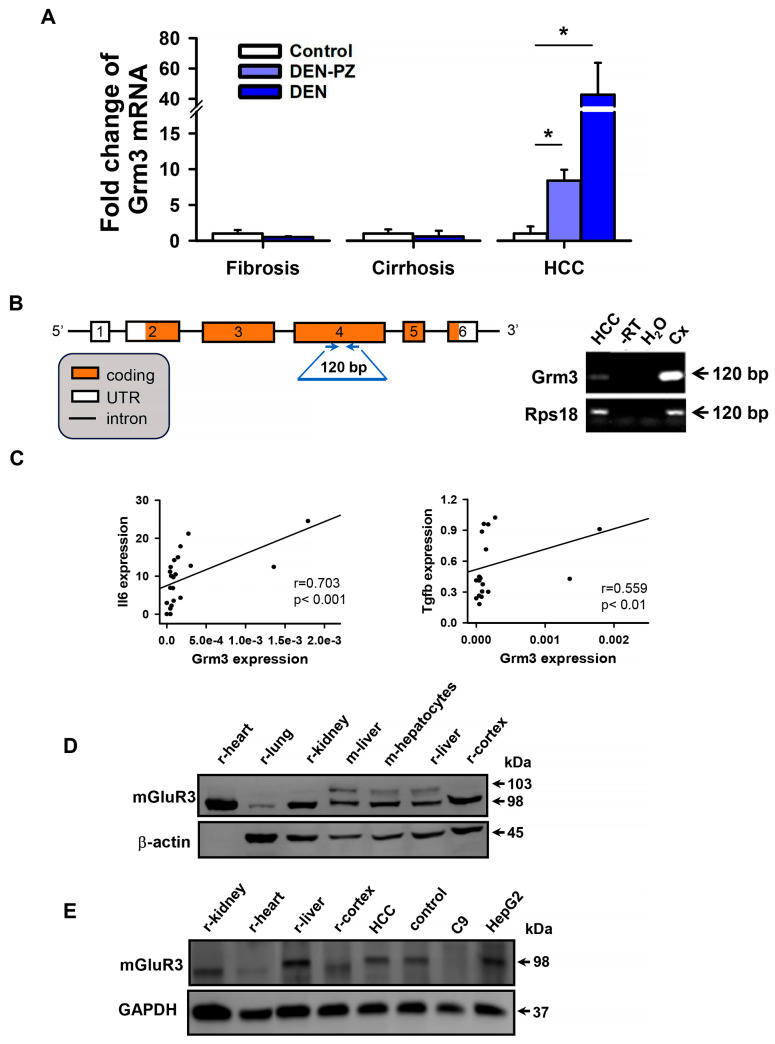
Liver mGluR3 expression and correlation of its mRNA levels with inflammation related-cytokines. Increased mGluR3 (*Grm3*) mRNA expression and a positive correlation with *Il-6* and *Tgfb* were observed in HCC. (**A**) *Grm3* expression in perfused livers of control, fibrosis, cirrhosis, and HCC rats was analyzed by RT-qPCR. In HCC, DEN-PZ indicates the peritumoral zone. The graph represents mean ± SEM (*n* = 5–12). Values were normalized to *Rps18* gene expression. * *p* < 0.05 by one-way ANOVA and Dunn’s post hoc test. (**B**) The identity of the *Grm3* qPCR product was confirmed by Sanger sequencing, and its size was verified by agarose gel electrophoresis. Exons of *Grm3* and the position of the oligonucleotides used are shown. (**C**) Spearman’s correlation plots between Grm3 and inflammation related-cytokines in HCC tissue samples with significant coefficients are shown. (**D**,**E**) Detection of the mGluR3 protein in different rodent tissues (r-rat, m-mouse). A band of the expected weight of ~98 kDa was detected in all tissues analyzed using anti-mGluR3 from (**D**) Abcam^®^ and (**E**) Alomone^®^. An additional band (~103 kDa) was observed in the liver and isolated hepatocytes, using Abcam^®^ antibody (HCC and control liver tissues obtained from the DEN model).

Studies have reported that mGluR3 is upregulated in macrophages in rats with CCl_4_-induced fibrosis and cirrhosis [17]. We investigated whether this enhanced expression would persist if the rats developed HCC. According to our findings, mRNA expression of *Grm3* tended to increase in cirrhosis and significantly increased in HCC both in tumoral and peritumoral tissues (Figure 4A). The identity of the qPCR product from liver *Grm3* mRNA was confirmed through Sanger sequencing (Figure 4B). The pattern obtained from Sanger sequencing was a 99% match with the publicly available rat mRNA sequence of the mGluR3 gene (*Grm3*) in NCBI (NM_001105712.1). Amplicons from the HCC tissue and cerebral cortex were noticeable in the agarose gel, confirming that the PCR product was identical in rat liver and brain (Figure 4B). To determine the potential effect of an inflammatory environment on the increment of *Grm3*, correlation between this mRNA and cytokine gene expression in liver tissue was evaluated using the Spearman test. A significant positive correlation was observed between *Grm3* and *Il6* and *Tgfb* (Figure 4C, Table S2).

To explore if the increased expression of *Grm3* was associated with the translated protein, liver tissue homogenates were processed by Western blot. We did not find any changes in band abundance in control, fibrosis, cirrhosis, or HCC liver tissues (Supplementary Figure S2). Interestingly, when comparing the liver with the cerebral cortex of rats, we observed a ~98 kDa band that co-migrated in both tissues. An additional ~103 kDa band was identified in the liver under all pathological conditions and in hepatocytes (Supplementary Figure S2), but not in other tissues, such as the heart, lung, kidney, or cerebral cortex (Figure 4D). The additional band could result from posttranslational modification of the protein in the liver, which could provide other properties to the protein. Given that no differences in protein expression were observed by Western blot, we used a different approach to analyze mGluR3 expression in the liver tissues.

### 2.4. Liver mGluR3 Protein Is Differentially Expressed in Pathological Stages Leading to HCC

**Figure 5:**
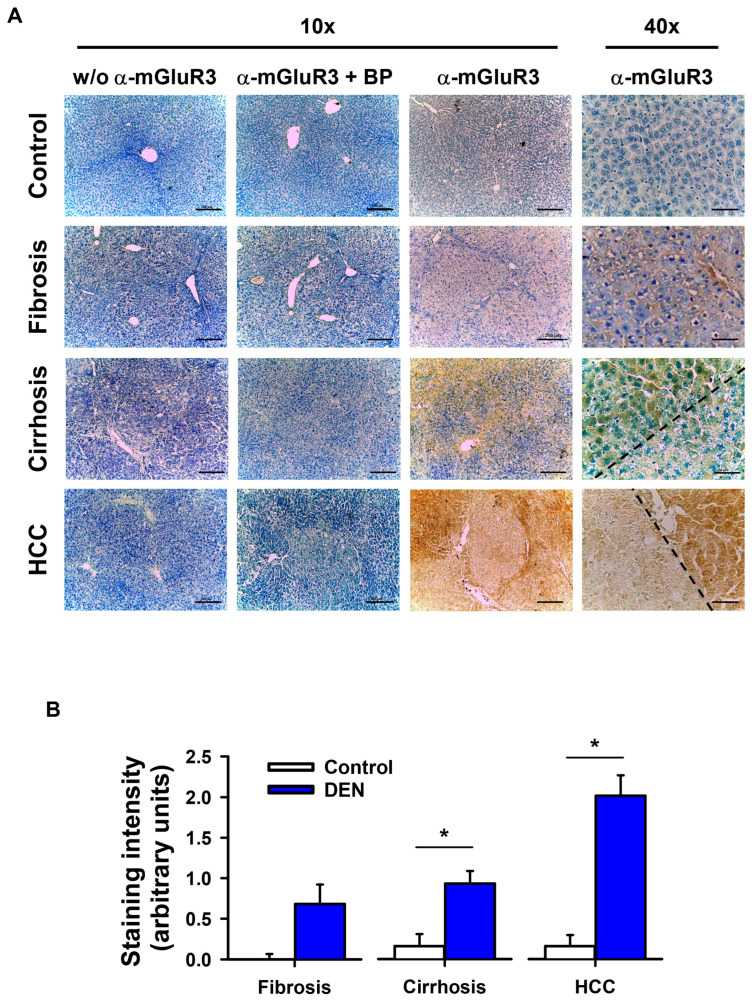
Liver immunodetection of mGluR3 during liver pathology progression in DEN-treated rats. (**A**) Representative images of mGluR3 staining (brown) and toluidine blue counterstaining of liver tissue from normal (control), fibrosis, cirrhosis, and HCC. The absence of primary antibody (*w*/*o*) or specific blocking peptide (BP) pre-incubated with anti-mGluR3 was used as a negative control. To the left of the dashed line, a dysplastic lesion associated with cirrhosis and a neoplastic lesion related to HCC are observed. Scale bar, 200 μm (10×) or 50 μm (40×). (**B**) Quantitative analysis was performed in ImageJ software. The graph represents quantification of mGluR3 by area in 10 high-power fields (40×). Data are expressed as mean ± SEM of three independent experiments, * *p* <  0.01 by *t*-test.

The presence of mGluR3 was analyzed using IHC in rat livers during the progression from fibrosis to cirrhosis to HCC induced by DEN. We identified a positive signal for mGluR3 under all conditions. This signal was corroborated as specific using a blocking peptide as a negative control (Figure 5A). Histological analysis showed that the positive signal increased as fibrosis progressed to HCC. Although staining for mGluR3 was heterogeneously distributed, we observed a discrete expression of mGluR3 in normal livers, which increased in fibrosis and became more evident in cirrhosis and HCC, in both tumor and peritumoral zones (Figure 5A). The quantitative analysis of staining was statistically significant in cirrhosis and HCC compared to normal liver (Figure 5B). The mean staining intensity of mGluR3 in the tumor and peritumoral zones is represented in the HCC group (Figure 5B).

**Figure 6:**
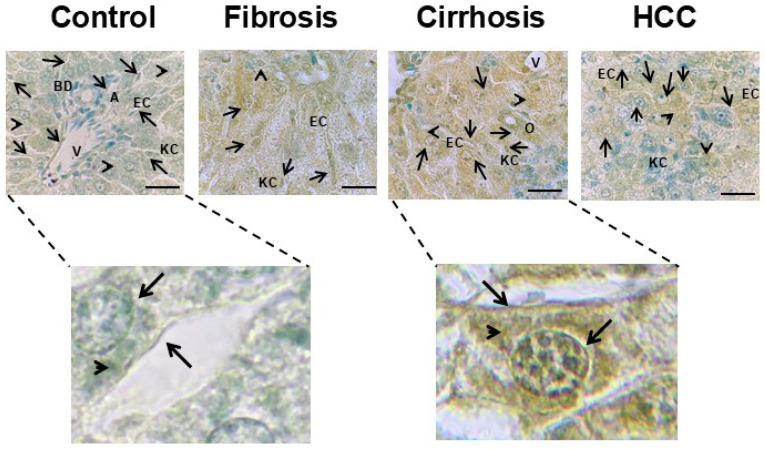
Cellular immunodetection of mGluR3 in HCC livers of DEN-treated rats. (**Upper panel**) Representative images of mGluR3 staining (brown) and toluidine blue counterstaining of liver tissue from normal (control), fibrosis, cirrhosis, and HCC. Scale bar, 30 μm (100×). Arrows indicate plasma or nuclear membrane, and arrowheads indicate the cytoplasmic location of mGluR3. (**Lower panel**) Zoomed-in areas are shown on the right. The control group shows a light positive signal for mGluR3 in intracellular locations, while the cirrhosis group exhibits a strong positive signal. Identification tags: A: artery, V: vein, O: hyperplastic oval cells, BD: bile ducts, KC: Kupffer cells, EC: endothelial cells.

IHC analysis of the liver tissue also provided detailed information about the cellular and subcellular location of the mGluR3 protein (Figure 6). Expression intensity was primarily assessed through direct visual observation using a qualitative scale, as shown in Table 1. The signal intensity in images was compared with the visual qualitative observation score: 0: No expression, +: Weak or very slight staining, ++: Moderate staining, +++: Strong staining. A positive signal for mGluR3 with different degrees of intensity was observed in the cytoplasmic membrane, cytoplasm, membrane nucleus, and nucleus in hepatocytes from normal and pathological livers (Figure 6, Table 1). Additionally, an analysis of the percentage of positive areas, expressed as the mean ± SD of 10–20 fields in two rats per group, showed increasing mGluR3 expression from control to fibrosis, cirrhosis, and HCC. In addition to hepatocytes, mGluR3 immunoreactivity was observed in non-parenchymal cells, including those identified as Kupffer and sinusoidal endothelial cells, based on morphological and histological criteria. Kupffer cells were identified by their localization within the sinusoidal lumen and characteristic morphology, including a rounded to irregular shape and prominent cytoplasm, whereas sinusoidal endothelial cells were recognized by their elongated nuclei lining the vascular spaces [27,28].

### 2.5. mGluR3 Protein Is Expressed in Normal Hepatocytes and HCC-Derived Cell Lines

**Figure 7:**
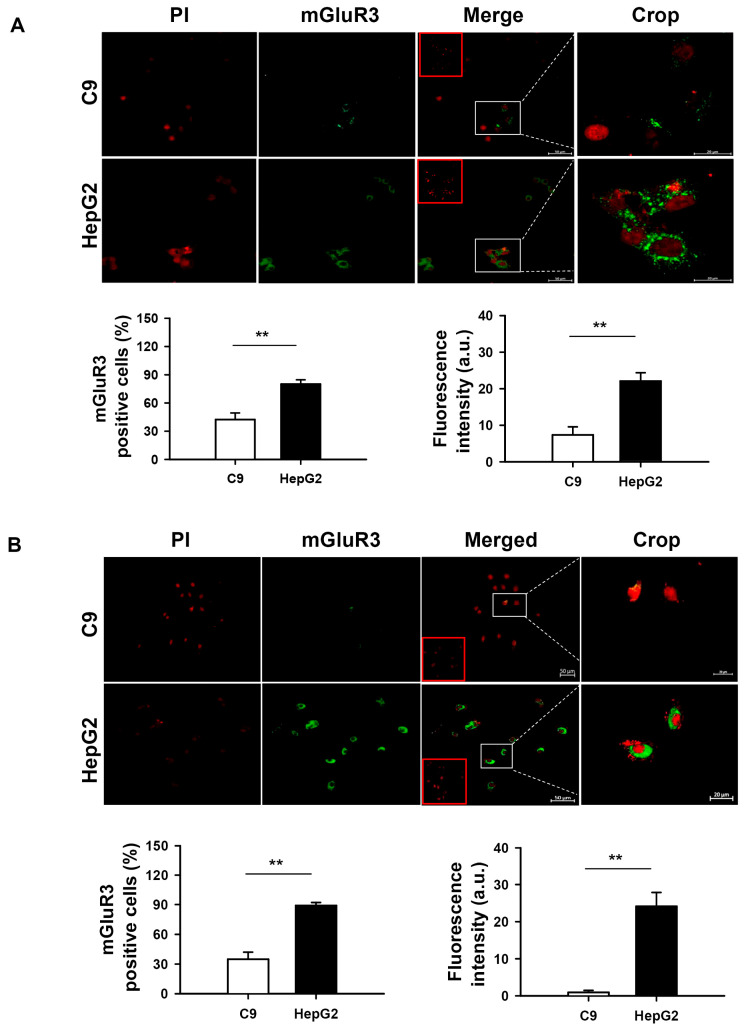
Immunodetection of mGluR3 in liver cell lines. Immunofluorescence staining for mGluR3 (green) and nuclei (red, propidium iodide) in C9 (rat normal hepatocytes) and HepG2 (human HCC cells); the merge of both channels is displayed. Immunostaining with anti-mGluR3 (**A**) Abcam and (**B**) Allomone antibodies is shown. Scale bar = 50 μm (25×) and 20 μm (crop). Crops of representative cells (white squares) are shown on the right. Negative controls in the absence of a primary antibody are shown as inserts (red squares). Quantitative analysis was performed in ImageJ software. Graphs represent the percentage of mGluR3-positive cells, and the average fluorescence intensity of mGluR3 was measured in 9–20 fields. Data are expressed as mean ± SEM of three independent cultures, ** *p* <  0.01.

To corroborate the cellular location of mGluR3, immunofluorescence staining was performed in two hepatocyte cell lines using two different antibodies (Figure 7A,B). mGluR3 immunoreactivity displayed a dense stippling in the cytoplasm and cell periphery; notably, this signal was intense in the HCC-derived HepG2 cell line compared to the weak staining observed in normal C9 hepatocytes (Figure 7). Videos of 3D projection show the subcellular distribution of mGluR3 in C9 (Video S1) and HepG2 (Video S2) cell lines. The enhanced mGluR3 signal and the number of positive HepG2 cells were detected with two different anti-mGluR3 antibodies, as shown in the graphs (Figure 7A,B, lower panels). The signal was absent in the negative control (no primary antibody).

### 2.6. mGluR3 Is Functional in HCC Cells

**Figure 8:**
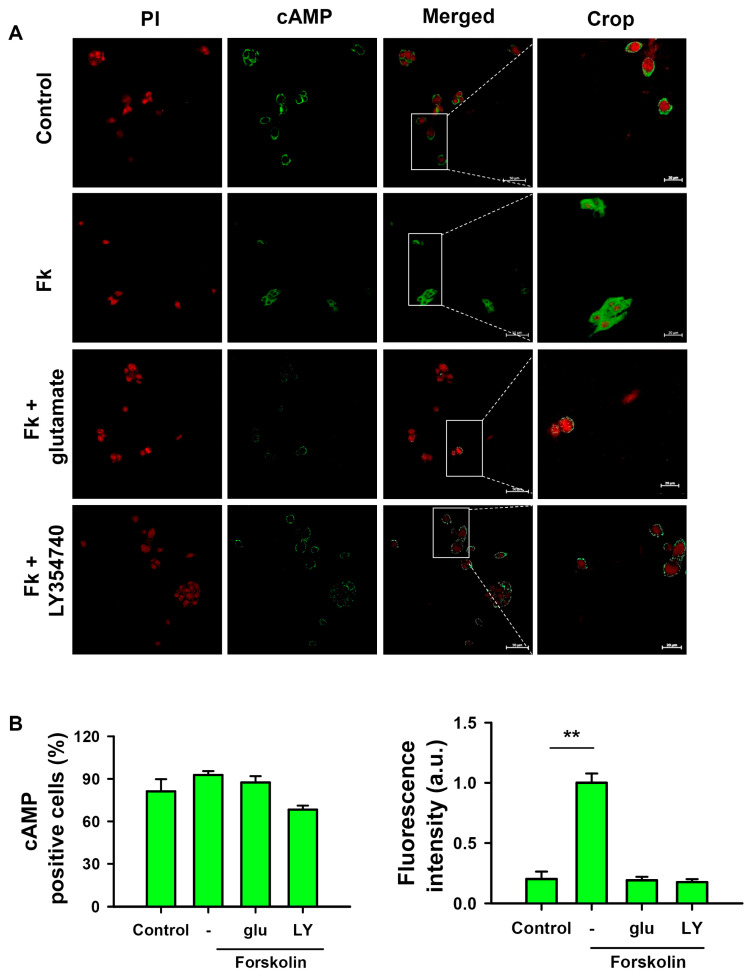
Effect of mGluR3 activation by glutamate or LY354740 on cAMP content induced by forskolin in HepG2 cells. (**A**) Immunofluorescence staining for cAMP (green) and nuclei (red, propidium iodide) in HepG2 cells; the merge of both channels is displayed. All cells displayed a positive cAMP signal, with different levels depending on the treatment. cAMP was enhanced by the adenylate cyclase activator forskolin (Fk, 10 μM) that was inhibited by glutamate (glu, 10 μM) or the mGluR3 selective agonist LY354740 (LY, 40 nM). Scale bar = 50 μm (25×) or 20 μm (crop). (**B**) Quantitative analysis of the number of positive cells and fluorescence intensity of cAMP measured in 10 fields. Data are expressed as mean ± SEM of three independent cultures. ** *p* <  0.01.

mGluR3 is a well-recognized receptor coupled to cAMP inhibition [29]. To assess the functional relevance of mGluR3 activation, we tested the effects of glutamate and group II-selective agonist LY354740 [30] on cultured HepG2 cells. We observed that pre-incubation with glutamate (10 mM) or LY354740 (40 nM) inhibited cAMP formation induced by the adenylate cyclase activator forskolin (10 mM), measured by immunofluorescence cytochemistry (Figure 8A). We also measured the signal intensity of cAMP-positive cells. Around 80–90% of cells quantified by nuclear PI staining (red) exhibited a cAMP-positive signal (green), independent of the treatment (Figure 8B, left graph). However, fluorescence intensity by cAMP was significantly diminished in response to the 1 h pre-incubation with glutamate or LY354740 upon stimulation with forskolin, indicating that mGluR3 is effectively activated by its ligands and is functional in terms of cAMP inhibition (Figure 8B, right graph).

### 2.7. mGluR3 Contributes to Survival in HCC Cells

**Figure 9:**
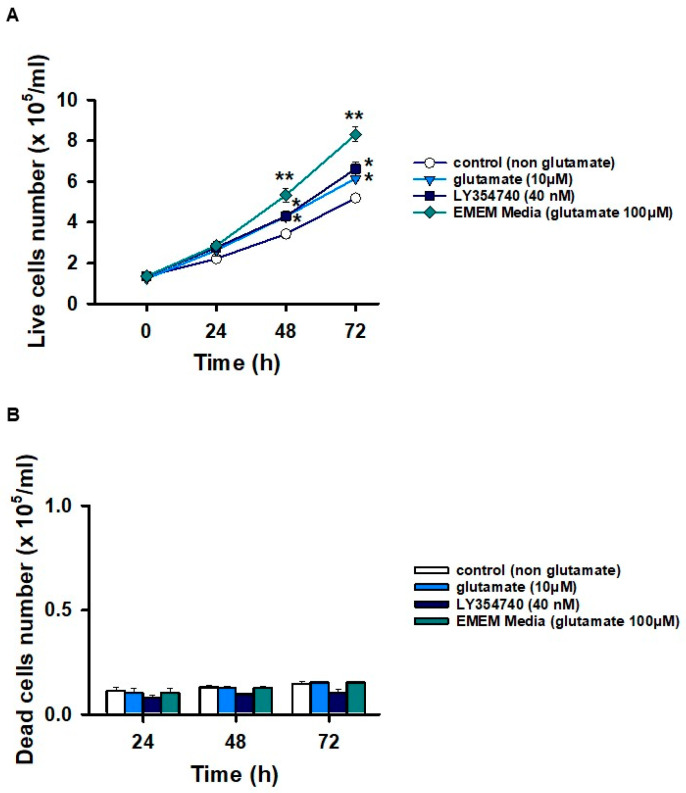
Time-course effect of mGluR3 activation by glutamate or LY354740 on viability in cultured HepG2 cells. (**A**) Time-course of viable cells, and (**B**) dead cells under treatment with or without mGluR3 agonists, visualized by trypan blue staining. The activation of mGluR3 by glutamate or LY354740 significantly influenced the viability of cultured HepG2 cells over time. Data are expressed as mean ± SD of five independent experiments; * *p* <  0.01; ** *p* <  0.05 vs. control by one-way ANOVA and Dunn’s post hoc test.

Once mGluR3 expression was clearly observed and its functionality demonstrated in HCC-derived cells, we investigated whether mGluR3 activation contributes to cell survival. To do so, we analyzed the effect of glutamate and the selective mGluR3 agonist LY354740. We observed that HepG2 cells did not die when incubated in a glutamate-free culture medium; however, they also did not grow, compared with cells incubated in the recommended EMEM media containing 100 mM glutamate (Figure 9A,B). Furthermore, treatment with a glutamate concentration that was one order of magnitude lower (10 mM) or with an ED_50_ of LY354740 (40 nM) over 24, 48, and 72 h resulted in a higher number of viable cells at 48 and 72 h of culture compared to cells without glutamate (Figure 9A).

## 3. Discussion

The present study indicates that liver mGluR3 expression is increased early in fibrosis and cirrhosis, and notably in HCC, as observed in a standardized rat model of hepatic disease progression induced by DEN. We demonstrate, for the first time, that liver mGluR3 expression is primarily localized in hepatocytes, both in tissue and cell lines. Additionally, mGluR3 immunoreactivity was observed in Kupffer cells and sinusoidal endothelial cells, as determined by morphological and histological criteria. Notably, the specificity of the immunohistochemical signal was supported by the use of a blocking peptide as a negative control. Furthermore, mGluR3 contributes to cell survival in HCC-derived cells, supporting a potential functional role in the context of tumor biology. The high concentration of serum and intrahepatic glutamate, the overexpression of xCT (Scl7a11, which exports glutamate), and the downregulated expression of EAAT2 (Slc1a2, which imports glutamate) in HCC livers suggest that glutamate exerts autocrine and paracrine actions by activating mGluR3.

The expression of mGluRs has been found in various tissues outside the central nervous system [2,31]. Some of these receptors have been identified as potential oncogenic drivers and may serve as biomarkers and therapeutic targets [32,33]. Moreover, abnormal glutamate signaling has been shown to play a role in the development, progression, and maintenance of various types of cancer, including melanoma, prostate, breast, and colon cancer [4,5,34,35]. In the liver, mGluR5 plays a significant role in liver injury and HCC [36,37]. Over 90% of HCC occurs in patients with underlying chronic liver diseases such as fibrosis and cirrhosis. The incidence of HCC continues to increase largely due to higher rates of metabolic disorders, the prevalence of viral hepatitis B and C, rising alcohol abuse, and the challenges in early diagnosis of the disease [14,38,39]. HCC has a high mortality rate primarily because it is diagnosed at a late stage and has limited therapeutic options. Therefore, early diagnosis of HCC is crucial, preferably before carcinogenesis occurs. Research has focused on the clinical utility of various serum and tissue markers for HCC diagnosis and prediction, including alpha-fetoprotein, glypican-3, golgi protein 73, vascular endothelial growth factor, Dickkopf-1, and midkine [40]. However, biomarkers are scarce and have limited diagnostic utility in detecting HCC [40]. Alpha-fetoprotein has been observed to correlate with aggressive disease and could serve as a predictor of treatment outcome, but it is limited in detecting small tumors [41,42,43,44]. In this study, we observed increased expression of both mRNA and protein levels of mGluR3 in different zones of the liver, including dysplastic nodules, neoplastic lesions, and peritumoral areas. This variation is part of the molecular heterogeneity associated with HCC development [45]. Furthermore, immunofluorescence analysis revealed significantly higher levels of mGluR3 protein in the HCC-derived cell line HepG2 compared to the normal hepatocyte cell line C9. These findings suggest that mGluR3 may serve as an early biomarker for HCC diagnosis.

The mRNA expression of mGluR3 in the liver is significantly lower than in the brain and is barely detectable in healthy rat livers, as measured by qPCR. However, under liver pathological conditions, its expression increases significantly as the disease progresses from fibrosis to cirrhosis and, ultimately, to HCC. Otherwise, the mGluR3 protein appears to be highly stable in the liver. While mGluR3 protein expression was identified by IHC in all stages, including healthy livers, it increased during the precancerous stage of cirrhosis and was highly overexpressed in HCC.

We observed a wide distribution of mGluR3 across the plasma membrane and subcellular compartments, such as the cytoplasm, nuclear membrane, and nucleus. Numerous plasma membrane receptors have been found in the nucleus as retrograde trafficking, which culminates in translocation into the nucleus, with consequences for cancer and other diseases [46,47,48,49]. In liver tissue, mGluR3, like other G protein-coupled receptors [50], displays cytoplasmic and nuclear localization, where it might exert a different function, such as regulating transcriptional activity [47]. In the HepG2 cell line, mGluR3 expression was detected in both the plasma membrane and the cytoplasm. It is well established that G-protein-coupled receptors can continue signaling after being internalized into endosomes. This process is regulated by diverse molecules, including those from the Ras family, as well as through posttranslational modifications [51]. Furthermore, G-protein-coupled receptors that undergo internalization after desensitization often recycle via endocytosis into early endosomes, which is a typical feature of these receptors [52]. The balance between endocytosis and recycling is crucial for maintaining plasma membrane composition, and disruptions of this balance can contribute to several pathologies, including cancer [53,54]. Studies showed that HEK293T cells transfected with mGluR3 (but not mGluR2) exhibited a recycling pattern characterized by rapid desensitization mediated by G-protein receptor kinases, followed by internalization via β-arrestins. This process involves trafficking and recycling back to the cell surface while minimizing lysosomal occupancy. These mechanisms depend on the doses of glutamate used; saturating doses resulted in enhanced receptor stability and reduced mGluR3 at the plasma membrane, whereas low doses led to transient coupling to kinases and less coupling to β-arrestin [55]. Recent research has proposed that activation and internalization of mGluR3 are differentially stabilized by orthosteric and allosteric ligands [56]. Our findings suggest that the translocation of mGluR3 into the nucleus is influenced by environmental signals that may vary in cultured cell lines. The mechanisms behind trafficking and its dependence on the agonists in the liver require further investigation. Additionally, cancer-associated mutations in the C-terminal domain of mGluR3 enhance β-arrestin coupling, leading to increased internalization [55]. Notably, elevated cytoplasmic and nuclear expression of G-protein-coupled receptors, particularly when associated with sustained active signaling, has been identified in various malignancies as a marker of poor prognosis and resistance to treatment [57]. Consequently, drug targeting to control both activation and desensitization, as well as addressing the impact of mutations, may influence the stability of receptors like mGluR3 and could lead to improved therapeutic strategies. Since degradation is not a primary downstream consequence of internalization, cells may not need active gene transcription, which is aligned with the low mRNA levels found in hepatic tissue and a high presence of mGluR3 in the cytoplasm, as observed with IHC and immunofluorescence staining.

Effective treatments for HCC represent a major challenge due to the tumor’s inherent heterogeneity, which is driven by genetic, proteomic, metabolic, and cellular diversity [58,59]. Therefore, understanding the molecular mechanisms of HCC initiation and development is crucial for developing accurate early diagnostic tools and efficient treatments.

The lack of concordance between mGluR3 mRNA levels and total protein abundance detected by Western blot highlights an important aspect of its regulation during disease progression. Both immunohistochemistry and immunocytochemistry revealed increased mGluR3 immunoreactivity in perfused liver, suggesting that its regulation may primarily occur through changes in cellular distribution and tissue compartmentalization rather than total protein expression. This is particularly relevant in the liver, a highly heterogeneous organ in which alterations in specific cell populations can be masked in whole-tissue homogenates. In this context, mGluR3 expression may resemble that of alpha-fetoprotein, which, although widely used as a biomarker, shows variable expression and limited sensitivity when used alone [60]. Accordingly, our findings open new avenues for investigating mGluR3 as a marker that may benefit from integrative approaches considering cellular context.

Although Western blot analysis revealed no differences in the abundance of the mGluR3 band in liver homogenates that co-migrated with brain homogenates (~98 kDa), an additional band migrating at a higher molecular weight (~103 kDa) was observed only in liver and isolated hepatocytes compared to other tissues, suggesting a posttranslational modification that might confer other properties, such as protein stability [61]. The tumor immune microenvironment, which consists of various cell populations and cytokines associated with chronic inflammation and that vary among patients, may influence the stability of several proteins. For example, IL-6 promotes the stability of programmed cell death-1 (PD-1) protein through deubiquitination, contributing to T-cell impairment and inhibiting its cytotoxic T-cell activity [62]. In our study, we found a positive correlation between the mRNA expression of mGluR3 and two cytokines (IL-6 and TGF-b) in the liver with HCC. The significance of this finding is still being investigated. Since TFG-b has been shown to provide stability to the mGluR3 protein in colon cancer [5], it is likely that it plays a similar role in liver tissue.

Our observations indicate that the liver expresses glutamatergic system components that enable glutamate signaling through a possible autocrine and/or paracrine circuit that accompanies cancer physiopathology, a consistent mechanism also observed in breast cancer [4]. We found that enhanced xCT and mGluR3 are associated with increased intrahepatic glutamate concentrations from fibrosis to HCC induced by DEN in rats. We consider that endogenous glutamate takes part in the biological actions of this receptor on HCC-derived cells since glutamate-free media were used to test the effects of activating mGluR3 with glutamate or the selective agonist LY354740. The absence of glutamate resulted in slower growth of HepG2 cells while maintaining cell viability, either by resisting cell death or enabling proliferative signaling [63]. Since exacerbated glutaminolysis generates high levels of glutamate from glutamine in cancer [4], it would be interesting to address the balance between glutamine synthase and glutaminase activities, as well as the effects of blocking glutamate exit transporters, such as xCT, to observe the endogenous actions over the different types of glutamate receptors in the liver.

Components of the glutamatergic system have been proposed as biomarkers with therapeutic value in oncogenic diseases [2]. Finding serum and tissue biomarkers to detect early-stage tumors in high-risk populations (e.g., patients with cirrhosis) could help in the diagnosis, prognosis, and implementation of more accurate treatments for patients with HCC, and therefore could lead to higher surveillance rates [38]. In some developing countries, identifying two or more biomarkers and conducting imaging studies improves the accuracy of diagnosis in high-risk individuals developing new or suspicious liver nodules [64]. Since the risk of HCC depends on the carcinogenicity of the underlying disease, carrying out risk-based surveillance for HCC in patients enables treatment at earlier stages and therefore reduces the risk of death. Hence, selecting the right individuals for clinical scrutiny is crucial to avoid overdiagnosis [39], which could lead to overtreatment, elevated costs, and adverse physical and psychological effects [38]. Moreover, detection of HCC in very early stages could lead to more options for curative therapies and, consequently, better HCC outcomes [12]. Further studies are needed to identify clinically valuable biomarkers. In this regard, components of the glutamatergic system, such as receptors and transporters, may improve diagnosis and serve as therapeutic targets in a multidisciplinary approach to treatment.

Regarding the limitations of this study, our findings suggest an association between mGluR3 expression and disease progression; however, they do not allow conclusions regarding causality. The underlying mechanisms still require further investigation. In addition, the discrepancy observed between mRNA and protein abundance, as assessed by Western blot, represents a limitation. Although this difference may reflect changes in cellular localization and tissue compartmentalization rather than overall protein levels, this interpretation remains speculative and requires further validation using quantitative protein-level approaches. While the experimental rat model effectively replicates key features of liver disease progression, it does not fully represent the complexity and heterogeneity of human HCC. Furthermore, the tumor microenvironment in human HCC, including the complexity of the immune cell dynamics, may influence mGluR3 expression and signaling, which deserves further study. The use of cell lines may not fully reflect the cellular interactions present in vivo. Finally, although mGluR3 emerges as a potential therapeutic target, additional preclinical and clinical validation is necessary.

## 4. Materials and Methods

### 4.1. Reagents

Diethylnitrosamine (DEN), (N0756), glutamate assay kit (MAK330), Minimum Essential Medium Eagle (MEM) (M0643), F12 Ham Kaighn’s Modification (F12K) (N3520), LY354740 (L1045), glutamate (L-glutamic acid monosodium salt, G5889), propidium iodide (P4170) were purchased from Sigma-Aldrich, St. Louis, MO, USA; Dulbecco’s Modified Eagle Medium (DMEM) low glucose from Gibco (Waltham, MA, USA); polyclonal rabbit anti-mGluR3 antibody (#AGC-012), mGluR3 blocking peptide (BLP-GC012) from Alomone (Jerusalem, Israel); polyclonal rabbit anti-mGluR3 antibody (ab140741), biotinylated goat anti-rabbit IgG antibody (ab6720), peroxidase–conjugated donkey anti-rabbit IgG antibody (AB97061) from Abcam (Cambridge, UK); monoclonal mouse anti-cAMP antibody (MAB2146) from R&D (Minneapolis, MN, USA); monoclonal rabbit anti-GAPDH (14C10), monoclonal rabbit b-actin (D6A8) from Cell Signaling Technology (Danvers, MA, USA); forskolin (1099) from Tocris (Minneapolis, MN, USA); RevertAid First Strand cDNA Synthesis Kit, Maxima SYBR Green qPCR Master Mix from Thermo Scientific (Waltham, MA, USA); TRIzol™ reagent, Alexa Fluor-488 donkey anti-rabbit IgG (A-21206), Alexa Fluor-488 goat anti-mouse IgG (H + L) (A-11001) from Invitrogen (Waltham, MA, USA); Vectashield^®^ (H1000), Vectastain Elite ABC-HRP kit (PK-6100), DAB Substrate Kit, Peroxidase (HRP), (3,3′-diaminobenzidine) (SK-4100) from Vector Laboratories (Newark, CA, USA); Direct-zol™ Miniprep RNA kit from Zymo Research (R2050) (Irving, CA, USA); AP conjugate substrate kit (1706432), protein assay dye (5000006) from Bio-Rad (Hercules, CA, USA).

### 4.2. Animals

All experiments were performed in accordance with international standards for the care and use of laboratory animals and approved by the Institutional Animal Care and Use Committee at the Instituto de Neurobiología, Universidad Nacional Autónoma de México (UNAM) (ethical approval code 084.A). Male Wistar rats (200 ± 20 g) were housed under 12 h light/12 h dark cycles, with lights on at 08:00 h, at a constant temperature (22 ± 1 °C), and with free access to water and standard rat chow (Laboratory Rodent Diet 5001, LabDiet, St. Louis, MO, USA).

### 4.3. Experimental Protocol of Liver Diseases

Animals were randomly assigned to different groups of four rats each. Experimental animals received weekly intraperitoneal injections of DEN (50 mg/kg of body weight) or 0.9% saline solution for 8, 12, and 16 weeks to induce fibrosis, cirrhosis, and HCC, respectively [22,65]. Body weight and food consumption were tracked throughout the experiment. At the end of each period, animals were euthanized by decapitation as follows: rats in the fibrosis and cirrhosis groups were sacrificed one week after the last DEN injection, whereas rats in the HCC group were sacrificed two weeks after the final injection, and samples of liver and trunk blood were collected. Blood was collected to analyze biochemical markers of liver injury and glutamate. Livers were dissected, weighed, and frozen at −80 °C to evaluate mRNA, protein, and glutamate expression, or fixed in 10% buffered formaldehyde solution for histological analysis and immunohistochemistry (IHC). To eliminate the possible influence of blood cells, four animals from each group were perfused with 0.9% sodium chloride solution through the portal vein, and liver expression of mGluR3 was analyzed using IHC.

### 4.4. Magnetic Resonance Imaging (MRI)

Animals were anesthetized with a 4–5% isoflurane/air mixture and a 2% mix to maintain anesthesia during image acquisition. Body temperature was maintained by recirculating warm water underneath the imaging bed, and vital signs were continuously monitored. Anesthesia was discontinued upon completion of the imaging session. A 5 min fast low-angle shot (FLASH) scan (without fat sat) was acquired with the following parameters: TR = 281 ms, TE = 2.6 ms, FOV = 64 × 64 mm^2^, slice thickness = 1 mm, no inter-slice gap, image size = 256 × 256, resolution = 0.253 mm, 26 slices, 4 averages. Imaging acquisition was around 10 min per animal due to respiratory gating.

### 4.5. Enzyme Quantification

Serum levels of aspartate transaminase (AST), alanine aminotransferase (ALT), alkaline phosphatase (ALP), lactate dehydrogenase (LDH), gamma-glutamyl transpeptidase (GGT), and total bilirubin were measured by an automatic analyzer (Medical Laboratory Chopo, Querétaro, Mexico).

### 4.6. RT-qPCR

mRNA expression of mGluR3, xCT, excitatory amino acid transporter 2 (EAAT2), cytokines IL-1b, IL-6, tumor necrosis factor-a (TNF-a), IL-10, and TGF-b, and collagen type 1 were analyzed by RT-qPCR. Total RNA extraction from 30 mg of liver tissue was performed using TRIzol™ reagent according to the manufacturer’s instructions. Then, total RNA purification was conducted using Direct-zol™ Miniprep RNA kit, following the manufacturer’s instructions. cDNA was synthesized from 2 ug of RNA using RevertAid First Strand cDNA Synthesis Kit. Reverse transcription and qPCR were performed using the CFX96^TM^ real-time PCR detection system (Bio-Rad, Hercules, CA, USA). Primers used for qPCR amplification were synthesized by Sigma-Aldrich Co. (St. Louis, MO, USA). Primer sequences, dilutions, and annealing temperatures are listed in Table S1. Amplifications were performed with Maxima SYBR Green qPCR Master Mix in a 10 μL final reaction volume containing diluted cDNA and 0.5 mM of each primer in SYBR Green qPCR Master Mix. Data were analyzed using the 2^−ΔΔCT^ method, and cycle thresholds were normalized to the Rps18 housekeeping gene.

### 4.7. Sanger Sequencing

Samples that were positive by RT-qPCR were visualized using 2% agarose gel electrophoresis and further amplified with the Sanger method using the sequencing service of the Proteogenomic Unit (Instituto de Neurobiología, UNAM). Sequencing data were analyzed using the UCSC Genome Browser.

### 4.8. Glutamate Quantification

Serum and hepatic content of glutamate were measured in duplicate by spectrophotometry using a glutamate assay kit according to the manufacturer’s instructions. Samples were previously deproteinized by adding an equal volume of ice-cold 1M perchloric acid; then, they were centrifugated at 10,000× *g* for 10 min at 4 °C. The collected supernatants were adjusted to pH 7.2 and stored at −20 °C for further quantification of glutamate.

### 4.9. Cell Lines

In this study, two representative hepatocyte cell lines were used: the normal rat hepatocyte Clone 9 or C9 cell line and the HCC-derived HepG2 cell line, which were kindly donated by Dr. Julio Pérez-Carreón from the Laboratory of Liver Diseases at the National Institute of Genomic Medicine, INMEGEN in Mexico City, Mexico. Both cell lines exhibit an adherent epithelial phenotype. C9 and HepG2 cells were cultured according to ATCC recommendations in F12-Ham (F-12K) and EMEM, respectively, supplemented with 10% fetal bovine serum (FBS), 1% penicillin/streptomycin, and 0.5% amphotericin. Cell cultures were maintained at 37 °C in a humidified atmosphere of 95% air and 5% CO_2_ in 75 cm^2^ cell culture flasks. Viable cell numbers were determined by trypan blue exclusion. All experiments were conducted with cultures below passage 19.

### 4.10. Histological Assessment

Liver tissue samples were fixed in 10% buffered formalin for 48 h at room temperature and dehydrated in a gradient of alcohols at increasing concentrations for paraffin embedding. Serial slices (5 mm thick) were obtained with an RM2135 microtome (Model RM2135, Leica Biosystems, Nussloch, Baden-Württemberg, Germany), then deparaffinized and stained with hematoxylin and eosin or Masson’s trichrome.

To evaluate the presence of mGluR3 in the liver, 5 μm thick paraffin sections were cut and then were deparaffinized, rehydrated, and permeabilized with citrate/Triton X-100 buffer (0.1% sodium citrate, 0.1% Triton X-100) for 8 min at room temperature, then slides were subjected to antigen retrieval in sodium citrate buffer (10 mM tri-sodium citrate, 0.05% Tween 20, pH = 6) for 2 min at 95–100 °C. After blocking with normal goat serum (1/20), sections were incubated overnight with the rabbit anti-mGluR3 antibody (Alomone) at a 1/200 dilution at 4–8 °C; then, the tissues were incubated with biotinylated goat anti-rabbit IgG at a 1/500 dilution, and the reaction was developed by the avidin–biotin–peroxidase complex using the Vectastain Elite ABC-HRP kit, revealed with the DAB Substrate Kit, and counterstained with toluidine blue staining. Negative mGluR3 controls were prepared by pre-incubating anti-mGluR3 (Alomone) for 1 h with the mGluR3 blocking peptide according to the manufacturer’s instructions. Another negative control was performed without primary antibody; instead, 1× PBS was used. Digital images were captured with a Leica DM500 optic microscope (Wetzlar, Germany).

Quantification of DAB-positive areas was performed on IHC sections using ImageJ software (version 1.2; W.S. Rasband, NIH, Bethesda, MD, USA). Images were acquired at 40× magnification using an Olympus CX23 microscope under identical exposure conditions. For each condition (control, fibrosis, cirrhosis, and HCC), 10–20 representative fields were captured from two independent experiments, avoiding areas with artifacts or sectioning defects. A standardized analysis pipeline was applied for all images, including color deconvolution and thresholding using Fiji/ImageJ. Threshold parameters were defined using negative controls, including sections incubated with mGluR3-blocking peptide, and consistently applied across all experimental conditions. Quantification was performed in a double-blinded manner, as previously described [66]. In addition, histopathological evaluation of staining patterns and identification of hepatic cell populations were independently performed by a trained pathologist.

### 4.11. Immunocytochemical Assessment for mGluR3 and cAMP

For mGluR3 immunodetection, HepG2 and C9 cells were seeded (1 × 10^5^ cells/well) on glass coverslips in a 12-well culture plate and cultured overnight. Then, the culture medium was removed and cells were fixed with 4% (*w*/*v*) paraformaldehyde (PFA) for 20 min at room temperature (RT), followed by three washes with 1× PBS pH 7.4, treated with 50 mM ammonium chloride (NH_4_Cl) for 30 min at RT, washed three times with PBS, and permeabilized (0.1% Triton X-100 in PBS) for 5 min. Next, cells were blocked with 5% *w/v* BSA in PBS for 1 h at RT, washed thrice with PBS, and incubated overnight at 4 °C with the respective rabbit anti-mGluR3 polyclonal antibody (Abcam or Alomone) at a 1/100 dilution in PBS; negative controls were incubated in PBS without the primary antibody. The following day, cells were washed three times with PBS and labeled with the secondary fluorescent conjugate Alexa Fluor-488 donkey anti-rabbit IgG at a 1/100 dilution in PBS containing 0.05% Tween-20 (PBST) for 1 h at RT. Finally, after two washes with PBS, cells were incubated with RNase (1/1000) for 5 min, counterstained with propidium iodide (5 mg/mL) for 10 min at RT, followed by three more washes with PBS, and mounted onto a slide using Vectashield^®^, sealed with transparent nail polish, and stored at 4 °C before imaging.

To test the effect of mGluR3 activation on intracellular cAMP content, HepG2 cells were seeded (3.5 × 10^4^ cells/well) on glass coverslips set in a 12-well culture plate and incubated for 60 min in DMEM under the same culture conditions. Then, the culture medium was removed with non-attached cells, and attached cells were pre-incubated with glutamate (10 mM) or the selective agonist LY354740 (40 nM) [30] for 1 h. Afterward, cells were treated with forskolin (10 mM) for 30 min. Then, the culture medium was retired, and cells were washed with 1× PBS (pH 7.4), then fixed with 4% PFA for 20 min at RT. PFA was removed, and cells were washed three times with 1× PBS (pH 7.4), treated with 50 mM NH_4_Cl for 30 min at RT, washed three times with PBS, and permeabilized (0.1% Triton X-100 in PBS) for 12 min. Next, cells were blocked using 5% (*w*/*v*) non-fat dry milk for 1 h at RT, washed thrice with PBS, and incubated overnight at 4 °C with the mouse anti-cAMP monoclonal antibody at a 1/150 dilution in Tris-buffered saline containing 0.05% Tween-20 (TBST). The following day, cells were washed three times with PBS and labeled with the secondary fluorescent conjugate Alexa Fluor-488 goat anti-mouse IgG at a dilution of 1/100 in TBST for 1 h at RT. To reduce nonspecific labeling, cells were incubated with RNase (1/1000) for 5 min, then cells were counterstained with PI (5 mg/mL) for 25 min at RT, followed by three more washes with PBS and mounted using Vectashield^®^ onto a slide, sealed with transparent nail polish, and stored at 4 °C before imaging.

### 4.12. Image Acquisition and Quantitative Analysis

Samples were visualized in a vertical Zeiss LSM 780 DUO confocal microscope with a 25× Plan-Apochromat multi-immersion objective (Zeiss, Göttingen, Germany). The fluorescence intensity of mGluR3-positive cells was quantified with ImageJ2 software https://imagej.net/software/imagej2/, accessed on 10 October 2022) using the free-hand selection and analysis tools from the main menu. For the methods above, 9–20 fields from two independent experiments for each cell line were evaluated. Micrographs were obtained from 85 to 105 cells, corresponding to 5–7 fields per sample from three independent experiments performed in duplicate. The relative mean fluorescence intensity and the percentage of positive cells were quantified using ImageJ software. The total cell count was determined by marking whole cells that exhibited a well-defined nucleus, while the positive cell count corresponded to those identified through mGluR3 staining. Subsequently, the percentage of positive cells was calculated.

### 4.13. Viability Assay

To evaluate the effect of mGluR3 activation on cell viability, C9 and HepG2 cells were seeded at 1 × 10^5^ cells/mL in 12-well plates in low-glucose DMEM without glutamate, supplemented with MEM and F-12 in equal amounts. Cells were then treated in duplicate with glutamate (2.5, 5, or 10 μM) or with the selective mGluR3 agonist LY354740 (10, 20, or 40 nM). Assays were performed for 24, 48, and 72 h. Live and dead cells were quantified by trypan blue exclusion. C9 control cells were kept in F-12 medium, and HepG2 control cells in MEM media containing 100 and 200 μM glutamate, respectively.

### 4.14. Western Blot

mGluR3 was analyzed by Western blot in homogenates of rat and mouse tissues (liver, heart, lung, kidney, and cerebral cortex) and cell lysates (isolated mouse hepatocytes, HepG2, and C9 cells). Randomly selected areas (~100 mg) of healthy, fibrotic, and cirrhotic livers and livers with evident nodules or tumors were dissected and homogenized using a protease inhibitor cocktail (Roche Diagnostics, Manheim DE) in homogenization buffer A. Total protein content was quantified using the Bradford method, and equal amounts of protein (80 μg) were separated in 10% acrylamide SDS-PAGE gels. Proteins were transferred to activated PVDF membranes (Immobilon P, Merck Co., Cork, Ireland), blocked in 5% fat milk/Tris-buffered saline (TBS), and incubated overnight with anti-mGluR3 antibody (1/1000) in TBS plus 5% BSA at 4 °C. As controls, anti-GAPDH (1/3000) or anti-b-actin (1/1000) antibodies were used. After washing three times in TBS plus 0.1% Tween-20, membranes were incubated with peroxidase-conjugated donkey anti-rabbit IgG antibody (1/5000) for 2 h at RT and visualized using AP conjugate substrate kit according to the manufacturer’s instructions. Image Lab v. 3.0 software (Bio-Rad, Hercules, CA, USA) was used for densitometric quantification of the bands.

### 4.15. Statistical Analysis

Results were expressed as mean ± SEM. Statistical analysis and graphics were carried out using SigmaPlot v14.5 Software (Systat Software Inc., San Jose, CA, USA). Data normality was assessed using the Shapiro–Wilk test. Parametric data were analyzed using Student’s *t*-test or one-way analysis of variance (ANOVA), with Tukey’s post hoc test. Non-parametric data were analyzed by the Mann–Whitney U test or Kruskal–Wallis one-way ANOVA using Dunn’s post hoc test. To examine the association between mGluR3 gene expression and the cytokine environment, Spearman’s rank correlation analysis was used. Differences in means were considered statistically significant at *p* < 0.05, and 95% confidence intervals were calculated. The number of samples (*n*), representing independent biological replicates, varied depending on the experimental approach and is indicated in the corresponding figure legends. Most experiments were performed in three independent replicates.

## Figures and Tables

**Table 1:** Hepatic cellular and subcellular distribution by Immunohistochemistry in DEN rat model. Assignment of score were based on visual qualitative observation score: 0: No expression, +: Weak staining, ++: Moderate staining, +++: Strong staining. Percent of positive areas were calculated in 10–20 fields in 2 independent experiments.

Experimental Group	Cellular Staining	Subcellular Staining in Hepatocytes	Score	% Positive Area (Mean ± SD)
Control	Hepatocytes. EC from portal triad veins and arteries, central veins, and sinusoids (LSEC), KC, epithelial cells from biliary ducts (BD)	Cytoplasmic membrane, cytoplasm	0 - +	8.5 ± 2.5
Fibrosis	Hepatocytes. EC from portal triad veins and arteries, central veins, and LSEC, KC, BD	Cytoplasmic membrane, cytoplasm, nuclear membrane	+ - ++	20 ± 1.7
Cirrhosis	Heterogeneous staining, mainly in hepatocytes from lobular zone around tracts, EC from central veins, LSECs, KC, oval cells	Cytoplasmic membrane, cytoplasm, nuclear membrane, nucleus	++ - +++	44.4 ± 3.2
HCC	Heterogeneous staining mainly in transformed hepatocytes, ECs, LSECs, BD, oval cells	Cytoplasmic membrane, cytoplasm, nuclear membrane, nucleus	++ - +++	50.8 ± 1.4

## Data Availability

The original contributions presented in this study are included in the article/Supplementary Materials. Further inquiries can be directed to the corresponding author.

## Supplementary Materials

The following supporting information can be downloaded at: https://www.mdpi.com/article/10.3390/ijms27093878/s1.
