# Supplementary figures and images for: Metabotropic Glutamate Receptor 3 Expression During Liver Disease Progression: Association with Inflammation and Cell Viability in Hepatocellular Carcinoma
Source: Int J Mol Sci. 2026 Apr 27;27(9):3878. doi: 10.3390/ijms27093878 (PMC13164193; doi:10.3390/ijms27093878)

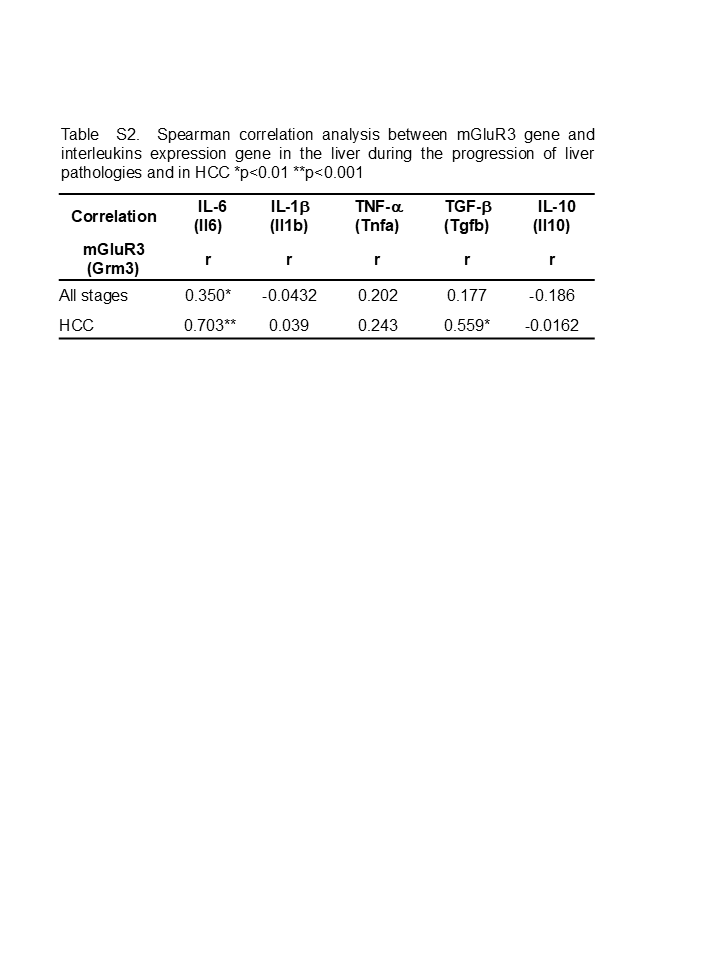

Supplement: Supplementary file 1 [file ijms-27-03878-s001.zip › Table S2. Spearman correlation analysis .tif]

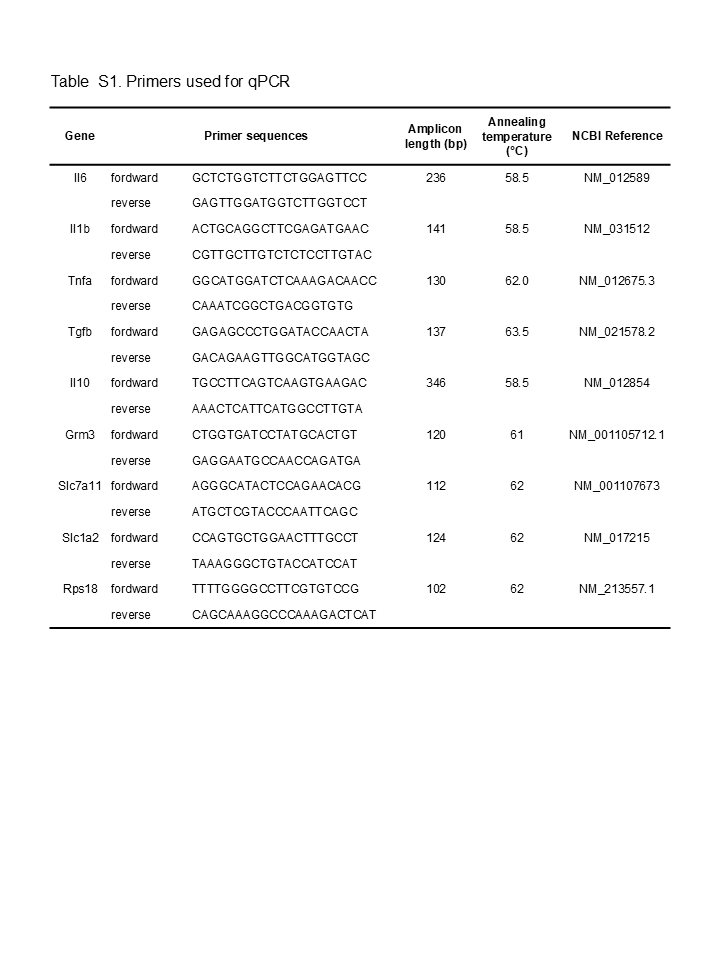

Supplement: Supplementary file 1 [file ijms-27-03878-s001.zip › Table S1. Primers used in PCR.tif]

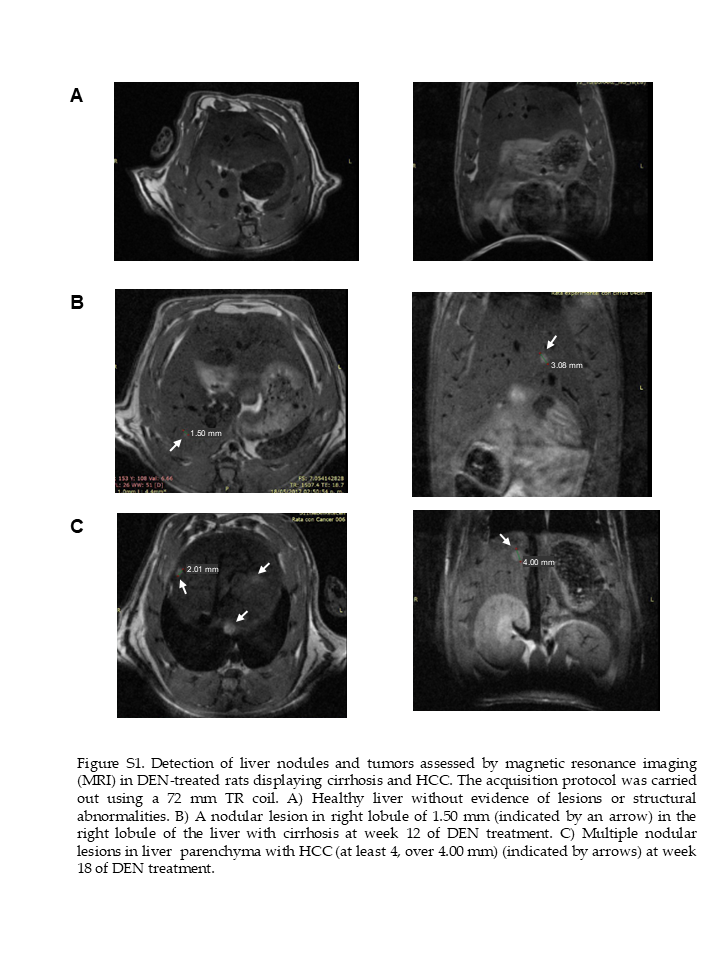

Supplement: Supplementary file 1 [file ijms-27-03878-s001.zip › Figure S1. Magnetic Resonance Image in cirrhosis and HCC.tif]

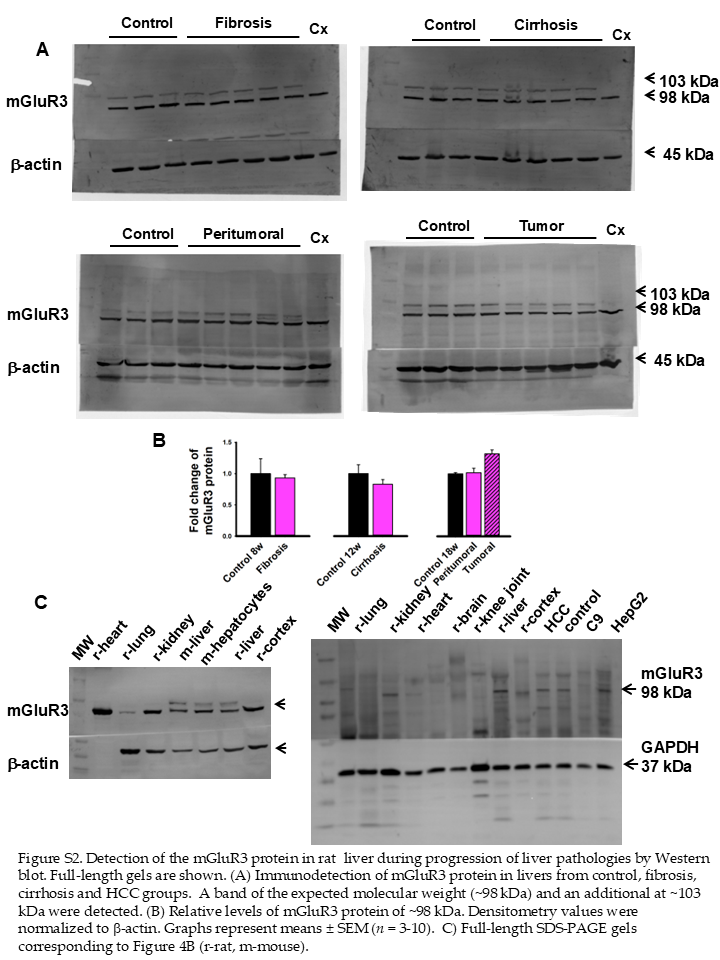

Supplement: Supplementary file 1 [file ijms-27-03878-s001.zip › Figure S2. mGluR3 by Western blot.tif]
